# Real-World Efficacy of COVID-19 Pre-Exposure Prophylaxis with Tixagevimab/Cilgavimab in People with Multiple Sclerosis

**DOI:** 10.3390/vaccines11121855

**Published:** 2023-12-15

**Authors:** Luke B. Elias, Aliya Jaber, Margarita Manzano, Mark Leekoff, Andrew Sylvester, Matthew A. Tremblay

**Affiliations:** Multiple Sclerosis Comprehensive Care Center, RWJBarnabas Health, Livingston, NJ 07039, USA; lukebennetelias@gmail.com (L.B.E.); mark.leekoff@rwjbh.org (M.L.); andrew.sylvester@rwjbh.org (A.S.)

**Keywords:** multiple sclerosis, tixagevimab, cilgavimab, COVID-19, Omicron

## Abstract

Vaccines against the SARS-CoV-2 virus were authorized for use by the Food and Drug Administration (FDA) in the United States and have proven effective for the prevention of morbidity and death from COVID-19. Certain immunosuppressant medications prevent the development of protective immunity following COVID-19 vaccination. In December 2021, the FDA issued an emergency use authorization (EUA) for a monoclonal-antibody combination of tixagevimab and cilgavimab, under the brand name Evusheld, for pre-exposure prophylaxis (PrEP) against COVID-19 for individuals with moderate-to-severe immune compromise. While a 77% reduction in symptomatic COVID-19 was observed in the PROVENT study, the trial was conducted prior to emergence of the B.1.1.529 Omicron variant. We suspected reduced efficacy of PrEP against Omicron subvariants. We conducted a retrospective cohort study comparing the prevalence of symptomatic COVID-19 infections between 1 January 2022 and 1 July 2022 in eligible patients treated with PrEP versus untreated using a questionnaire administered with the REDCap survey tool. Responses from 235 participants were included in the final analysis, with 176 untreated respondents and 59 in the PrEP cohort. Symptomatic COVID-19 infections were reported in 50 (28.4%) untreated participants and only 9 (15.3%) of those who received PrEP (*p* = 0.0557; OR 0.4536; 95% CI 0.2046 to 0.9599). Only two participants were hospitalized for COVID-19 infection, both in the untreated cohort. The reduction in COVID-19 infections did not achieve statistical significance, indicating diminished efficacy against Omicron variants.

## 1. Introduction

According to the World Health Organization (WHO), COVID-19 has claimed over 1.1 million lives in the United States and 6.8 million lives worldwide as of April 2023 [1]. In late 2020, two mRNA vaccines against SARS-CoV-2 were authorized for prevention of COVID-19 and have proven to be effective for the prevention of morbidity and death from COVID-19 [2,3]. Certain immunosuppressant medications prevent the development of protective immunity against COVID-19 [4,5,6]. Anti-CD20 monoclonal therapies are among the most-used disease-modifying therapies (DMTs) for the treatment of multiple sclerosis (MS), including ocrelizumab, ofatumumab, ublituximab, and off-label rituximab. These medications result in the dramatic muting of humoral immunity to novel antigens because they act by depleting circulating B-lymphocytes [7]. Many patients on anti-CD20 therapy failed to develop antibodies against SARS-CoV-2 following vaccination for COVID-19 [4,5,6]. Tixagevimab and cilgavimab were authorized for use in combination under the trade name Evusheld for pre-exposure prophylaxis (PrEP) to prevent symptomatic COVID-19 infections in people with moderate or severe immune compromise who were not expected to mount adequate immune responses to COVID-19 vaccines, as well as those with a severe allergy to COVID-19 vaccines. Their parent antibodies were derived from B-cells donated by convalescent patients after recovery from COVID-19 infection and subsequently engineered for longer half-lives [8]. In the PROVENT Phase III clinical trial, tixagevimab/cilgavimab resulted in a statistically significant reduction (77% at primary analysis, 83% at median six-month analysis) in the risk of developing symptomatic COVID-19 [8]. COVID-19 infection data from PROVENT included exposure to multiple variants of SARS-CoV-2 but preceded the emergence of the B.1.1.529 Omicron variant. Shortly after Omicron variants became the predominant strain of SARS-CoV-2 in the US, multiple studies demonstrated decreased neutralizing activity of tixagevimab and cilgavimab in vitro, ranging from a 12-fold-to-37-fold reduction [9,10] for the initial Omicron variant. The current study was conceived in response to observing multiple breakthrough COVID-19 infections in people with MS who received the full dose of tixagevimab/cilgavimab. Similar breakthrough COVID-19 infections were also reported in other clinical contexts [11]. Given the observation that multiple breakthrough infections were observed in patients treated by our MS center, our hypothesis was that tixagevimab/cilgavimab provided less protection against COVID-19 infection from Omicron variants than previous strains of SARS-CoV-2.

The goal of our study was to evaluate the efficacy of PrEP with tixagevimab/cilgavimab against Omicron subvariants in a real-world cohort. To do so, we performed a retrospective cohort analysis in people with MS (PwMS) on anti-CD20 therapy comparing the prevalence of symptomatic COVID-19 infections in those treated with PrEP compared to an untreated cohort.

## 2. Materials and Methods

### 2.1. Study Design

A retrospective cohort design was utilized to analyze the clinical efficacy of tixagevimab/cilgavimab. We selected a study period for analysis from 1 January 2022 through 1 July 2022 to include the first month in which PrEP was administered within our patient cohorts, a period in which the predominant SARS-CoV-2 strains were Omicron variants, and to align with the 6-month interval for re-dosing defined by the FDA label [12]. Eligible participants were contacted by telephone for voluntary participation in a brief survey. Participants were provided with an electronic consent waiver and a link to the study-specific REDCap survey. Basic demographic information, treatment history, and details of any COVID-19 infections during the study period were obtained. Cohorts were assigned based on exposure to tixagevimab/cilgavimab during the study period. Participants were assigned to the PrEP cohort regardless of the dose and date of administration unless (1) the initial tixagevimab/cilgavimab dose was administered after the study period or (2) the symptomatic COVID-19 infection reported during the study period occurred prior to administration of tixagevimab/cilgavimab. Participants who initiated PrEP after the study period or experienced symptomatic COVID-19 infections prior to PrEP were assigned to the untreated cohort. Participants were classified as having a COVID-19 infection during the study period if (1) symptoms were confirmed with a positive test, including home antigen testing, or (2) symptoms were concurrent with exposure to a household contact with a confirmatory test.

### 2.2. Study Participants

**Figure 1:**
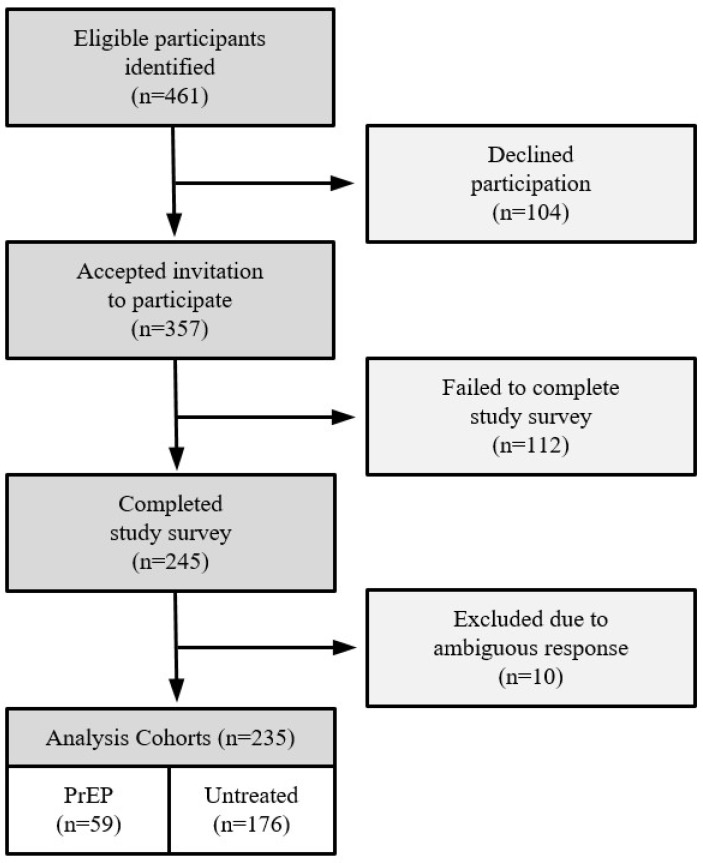
Study participant disposition.

All eligible participants were patients treated at a single MS center located in northern New Jersey, United States. The study team identified all patients at our center treated with anti-CD20 therapies (including ocrelizumab, rituximab, and ofatumumab) within 6 months of the study period. At study conception, we estimated that our center had administered PrEP to between 90 and 100 patients using anti-CD20 therapy to treat MS. A total of 461 eligible patients were identified based on treatment history and invited to participate in the study (Figure 1). In total, 357 requested to receive the REDCap survey, while the remaining 104 declined to participate. A total of 245 participants completed the questionnaire. In total, 10 subject records were removed due to ambiguity in responses and/or repeated entries by the same participant. Responses from 235 participants were included in the final analysis, 59 in the PrEP cohort and 176 in the untreated cohort. Two participants were re-classified as untreated because they reported a COVID-19 infection during the study period prior to tixagevimab/cilgavimab administration. A total of 13 subjects were reassigned from the PrEP cohort to the untreated cohort because their initial tixagevimab/cilgavimab exposure date was after the study period.

### 2.3. Data Analysis

Data obtained included limited demographic information; current DMT; vaccine status and timing; seroconversion status; date of tixagevimab/cilgavimab administration, as well as timing; severity; and treatment for any COVID-19 infections. Demographic data were aggregated, and descriptive statistics were performed. Rate of COVID-19 infections and other dichotomous variables were compared using the chi-square and Fisher’s exact tests. Analysis of continuous quantitative variables utilized *t*-tests, ANOVA, and corrections for multiple comparisons. Logistic regression modeling was used to assess the effect of age on the primary outcome. Based on the sample size of this study, we estimated that our analysis would be adequately powered to detect a 66% difference in the incidence of COVID-19 between treatment groups, which is below the 95% confidence interval for the relative risk reduction seen with a 6-month follow-up in the PROVENT study [65.8 to 91.4%] [8].

### 2.4. Ethics

This study was conducted according to local regulations and in compliance with the principles of the Declaration of Helsinki 1964. All applicable International Conference on Harmonisation guidelines were adhered to, and this study’s protocol was approved by the Institutional Review Board of Cooperman Barnabas Medical Center (Study Number 22-34). Informed consent was waived due to the minimal risk of this study. Eligible patients were provided with an electronic letter detailing the risks and benefits of this study as part of their invitation to participate and informed that completion of the survey implied their consent to participate in the study.

## 3. Results

### 3.1. Baseline Demographics

Some notable differences were detected between cohorts (Table 1). The mean age of participants in the PrEP cohort was greater than the untreated cohort. Participants in the PrEP cohort were also more likely to have received a COVID-19 vaccine. Specific details regarding type, brand, and number of vaccine doses are not reported due to the heterogeneous nature of the data. Many participants were immunized with multiple different COVID-19 vaccine products. Many obtained more than the recommended number of doses, in response to failed seroconversion, as a consequence of their DMTs. A significantly higher proportion of participants in the untreated cohort had a history of COVID-19 infection prior to the study period.

### 3.2. COVID-19 Infections

Symptomatic COVID-19 infections were reported in 50 (28.4%) untreated participants and only 9 (15.3%) of those who received PrEP (Table 2). The difference did not achieve statistical significance (*p* = 0.0557), suggesting that the efficacy of PrEP against the initial Omicron variants was below that reported in the PROVENT study. However, the odds ratio (OR = 0.4536; 95% CI 0.2046 to 0.9599) suggested a trend toward a protective effect that our sample size was likely too small to adequately detect. Only two participants were hospitalized for COVID-19, neither of whom received PrEP. Of the two hospitalized patients, only one required critical care admission for severe COVID-19. While this study was not powered to evaluate a treatment effect on the severity of COVID-19 infection, it is noteworthy that no severe infections were reported in the PrEP cohort.

### 3.3. Post Hoc Analyses

Shortly after the regulatory authorization of tixagevimab/cilgavimab for PrEP, the recommended dose was increased out of concern for decreased neutralization activity against Omicron variants of SARS-CoV-2. Consequently, many eligible recipients were advised to receive an additional dose. Of the 59 respondents who received PrEP, 27 (45.8%) specified the total dose of medication received. A total of 9 (33%) received only the initial 150 mg dose, while 18 (66.7%) completed the full updated 300 mg dose. Only one (11.1%) of the participants who received 150 mg doses reported symptomatic COVID-19, compared with five (27.8%) who completed the full 300 mg dose.

Additional analyses were used to address some of the confounding differences between treatment groups. To address the potential effect of differing mean age between the treatment groups, we fit a logistic regression model including both age and treatment. The effect of age was found to have a non-significant *p*-value (*p* = 0.0776), and thus, we report the *p*-value and odds ratio from the original Fisher’s exact test.

While vaccination was less common among the untreated cohort, we did not observe an effect on the risk of COVID-19. Symptomatic COVID-19 infections were reported in 7 (25.9%) unvaccinated participants and 52 (25.0%) vaccinated participants. Proportions infected with COVID-19 were also similar among recipients of different initial vaccine products, which are as follows: 4/12 (33.3%) for Janssen Ad26.COV2.S, 23/92 (25.0%) for Moderna mRNA-1273, and 25/104 (24.0%) for Pfizer/BioNTech BNT162b2. The similar risk of symptomatic COVID-19 in both vaccinated and unvaccinated individuals within the study population is likely due to lack of humoral immune responses to vaccine in individuals on anti-CD20 monoclonal therapy [4,5,6].

## 4. Discussion

The primary objective of this study was to evaluate the real-world efficacy of tixagevimab/cilgavimab against symptomatic COVID-19 following the emergence of the Omicron variant of SARS-CoV-2. Based on our observation of breakthrough infections and published in vitro data, our hypothesis was that PrEP with tixagevimab/cilgavimab would have diminished efficacy during the study period, compared to results of the PROVENT study. We did not observe a significant treatment effect of PrEP in a cohort adequately sized to detect a 66% relative risk reduction. The findings of our study suggest that PrEP was less effective against Omicron variants than strains of SARS-CoV-2 to which participants were exposed in the pivotal trial. While the difference in proportion of patients reporting symptomatic COVID-19 fell short of statistical significance, the odds ratio was similar to that in published studies, which did observe a statistically significant treatment effect and thus clinical efficacy of PrEP against Omicron variants [13,14].

A retrospective study demonstrated that PrEP decreased the risk and severity of symptomatic COVID-19 infection, during a period of predominantly Omicron subvariants, in a population of patients who had undergone allogeneic hematopoietic stem-cell transplantation and lacked humoral immunity to COVID-19 vaccine [15]. A similar study found a protective effect of tixagevimab/cilgavimab in rheumatology patients treated with rituximab [16]. Given that these studies were able to demonstrate such efficacy, our inability to demonstrate efficacy was likely a consequence of sample size and statistical power.

The main limitations of our study include a retrospective design, utilization of a patient-reported outcome, small sample size, and unequal distribution between cohorts. Based on cohort sizes, this study was adequately powered to detect a 66% difference in incidence of symptomatic COVID-19 infections between the PrEP and untreated cohorts. While there were notable imbalances in age and vaccination status between study cohorts, additional analyses suggest that neither variable was responsible for failure to observe a significant treatment effect. In fact, most of the symptomatic COVID-19 infections reported in our study occurred in vaccinated individuals.

Near the end of 2022, Omicron subvariants emerged for which tixagevimab/cilgavimab exhibited diminished neutralization activity [17]. Ultimately, on 26 January 2023, the authorization for emergency use of tixagevimab/cilgavimab was withdrawn by the FDA in response to expected loss of efficacy against current and future Omicron subvariants [18]. Availability of pre-exposure prophylaxis against COVID-19 remains an important option for immunocompromised populations and a continued area of relevance to numerous medical specialties, particularly transplant and oncology. Immunocompromised individuals remain disproportionately more impacted by COVID-19 than the general population. A recent retrospective cohort study found that immunocompromised individuals accounted for 22% of all COVID-19 hospitalizations during the Omicron era, despite representing only 3.9% of the study population [19]. Patients with solid-organ transplant, stem-cell transplant, and recent treatment for hematologic malignancy all reported over a 10-times greater risk of hospitalization and at least 6-fold greater risk of death from COVID-19, despite vaccination. Fortunately, there are newer monoclonal antibodies in development for PrEP against contemporary strains of SARS-CoV-2, including those being tested in the ongoing SUPERNOVA trial [20].

## 5. Conclusions

The findings of our study support our initial hypothesis that the emergence of the Omicron variant of SARS-CoV-2 resulted in reduced efficacy of PrEP with tixagevimab/cilgavimab. The trend toward the lower incidence observed in the PrEP cohort is aligned with that in published reports that demonstrated efficacy, albeit less than anticipated from the pivotal trial.

## Figures and Tables

**Table 1:** Demographic data of primary study analysis. PrEP and untreated cohorts are split to show differences and similarities. * A total of 2 patients in the PrEP cohort had non-MS diagnoses, and 26 participants did not specify their MS subtype.

Demographics			
	PrEP	Untreated	*p*-Value
***n***	59	176	
**Age, mean (SD)**	56.4 (11.0)	49.1 (12.1)	<0.0001
**Female, *n* (%)**	42 (71.2%)	124 (70.5%)	>0.9999
MS Subtype *, *n* (%)			
Relapsing	36 (66.7%)	116 (75.8%)	0.2116
Progressive	18 (33.3%)	37 (24.2%)	
**DMT, *n* (%)**			
Ocrelizumab	30 (50.8%)	120 (68.2%)	0.053
Ofatumumab	2 (3.4%)	5 (2.8%)	
Rituximab	27 (45.8%)	51 (29.0%)	
**Vaccination Status, *n* (%)**			
Vaccinated	57 (96.6%)	151 (85.8%)	0.0312
Unvaccinated	2 (3.4%)	25 (14.2%)	
**COVID-19 History, *n* (%)**			
Prior COVID-19	12 (20.3%)	72 (40.9%)	0.0046
Uninfected	47 (79.7%)	104 (59.1%)	

**Table 2:** Efficacy of PrEP with tixagevimab/cilgavimab. Difference in the risk of symptomatic COVID-19 infections indicates a trend that fell below the threshold of statistical significance (*p* = 0.0557), while odds ratio suggests a protective effect (OR = 0.4536; 95% CI 0.2046 to 0.9599). Hospitalization and ICU admission were only seen in the untreated cohort.

	PrEP (*n* = 59)	Untreated (*n* = 176)	*p*-Value OR (95% CI)
COVID-19 Infection	9 (15.3%)	50 (28.4%)	0.0557
No Infection	50 (84.7%)	126 (71.6%)	OR = 0.4536 (0.2046 to 0.9599)
Hospitalization	0 (0%)	2 (4%)	NA
ICU Admission	0 (0%)	1 (2%)	NA

## Data Availability

Data are not publicly available but can be made available upon request to the corresponding author.
